# Vitamin D Sufficiency Revisited: Evidence of a Dose–Response Effect for MASLD in Adults at Risk

**DOI:** 10.3390/nu18040599

**Published:** 2026-02-11

**Authors:** Gediz Dogay Us, Francesco Innocenti, Ozgur Muhammet Koc, Volkan Demirhan Yumuk, Zeynep Banu Gungor, Ger H. Koek

**Affiliations:** 1 School of Nutrition and Translational Research in Metabolism, Maastricht University, 6211 LK Maastricht, The Netherlands; 2 Department of Gastroenterology, Pax Clinic, 34365 Istanbul, Turkey; 3 Division Endocrinology, Metabolism and Diabetes, Cerrahpaşa Medical Faculty, Istanbul University-Cerrahpasa, 34098 Istanbul, Turkey; 4 Department of Methodology and Statistics, CAPHRI Care and Public Health Research Institute, Maastricht University, 6211 LK Maastricht, The Netherlands; 5 Department of Gastroenterology and Hepatology, Maastricht University Medical Center, 6211 LK Maastricht, The Netherlands; 6 Department of Medical Biochemistry, Cerrahpaşa Medical Faculty, Istanbul University-Cerrahpasa, 34098 Istanbul, Turkey

**Keywords:** MASLD, fatty liver, vitamin D, VCTE, NAFLD

## Abstract

**Background and Aims:** Vitamin D plays a pivotal role in liver health, influencing multiple steps in the development of steatosis, fibrosis, and extrahepatic complications in metabolic dysfunction-associated steatotic liver disease (MASLD). However, serum vitamin D concentrations that confer optimal hepatic protection in MASLD remain unclear. We therefore aimed to investigate the association between vitamin D status and MASLD and to explore whether higher vitamin D concentrations confer incremental protection beyond current sufficiency cut-offs. **Method:** We conducted a multicenter cross-sectional study of 1039 adults with at least one cardiometabolic risk factor for MASLD diagnosis, recruited between 2022 and 2024. Participants that reported excessive alcohol intake (>30 g/day for men, >20 g/day for women) and other etiologies of liver disease were excluded. Serum vitamin D levels were measured, with ≥20 ng/mL defined as sufficiency. MASLD (controlled attenuation parameter [CAP] ≥ 248 dB/m) and significant fibrosis (liver stiffness measurement [LSM] ≥ 8 kPa) were assessed using vibration-controlled transient elastography. Missing vitamin D values were imputed with multiple imputation. Associations between vitamin D status, MASLD and fibrosis were examined using multivariable logistic regression models adjusted for potential confounders. **Results:** Participants had a mean age of 52.2 ± 13.0 years; 51.6% were male and mean BMI was 30.1 ± 5.8 kg/m^2^. Vitamin D sufficiency and obesity were present in 81.2% (95% CI: 78.4–84.9) and 54.7% (95% CI: 51.3–58.0), respectively. Vitamin D sufficiency was associated with lower odds of MASLD (crude OR = 0.47, 95% CI: 0.33–0.67) and significant fibrosis (crude OR = 0.46, 95% CI: 0.28–0.76). After adjusting for potential confounders, the association between Vitamin D sufficiency and MASLD remained clinically relevant but did not reach statistical significance (adjusted OR = 0.60, 95% CI: 0.36–1.03, *p* = 0.06). In contrast, the association between Vitamin D sufficiency and significant fibrosis was both clinically relevant and statistically significant (adjusted OR = 0.48, 95% CI: 0.246–0.916, *p* = 0.03). When Vitamin D was categorized into quartiles, participants in the highest quartile (≥44 ng/dL) had 61% lower odds of MASLD in the adjusted model (adjusted OR = 0.39, 95% CI: 0.21–0.71) compared with participants in the lowest quartile (≤22 ng/mL). No significant dose-dependent associations were observed for fibrosis. **Conclusions:** Vitamin D levels showed a dose-dependent decrease in the odds of MASLD among at-risk adults. While the protective effect on fibrosis was not dose-dependent, these findings collectively suggest vitamin D as a potentially modifiable factor in MASLD prevention and management.

## 1. Introduction

Vitamin D, a pleiotropic hormone best known for its role in bone homeostasis, exerts important extraskeletal effects on systemic metabolism. Through multiple biological pathways, vitamin D influences glucose and lipid regulation, inflammation, and immune function. Accordingly, low circulating serum vitamin D levels have been linked to insulin resistance, type 2 diabetes, cardiovascular disease, and metabolic syndrome [1,2]. More recently, growing interest has focused on the potential role of vitamin D in the development of metabolic dysfunction-associated steatotic liver disease (MASLD). With its regulatory effects on adipose tissue function, inflammation, and fibrogenesis, vitamin D status may contribute to MASLD development and progression [3].

Due to the liver’s role in metabolizing vitamin D, suboptimal vitamin D levels may be expected in liver dysfunction. Indeed, vitamin D insufficiency has frequently been reported in individuals with MASLD compared to healthy controls [4,5]. However, findings on the bi-directional association between vitamin D and MASLD are conflicting, as some studies have shown an independent relationship [6,7], while others have not [8,9]. These inconsistencies likely stem from methodological constraints, such as small sample sizes [10,11,12] and reliance on serum transaminases, which are insensitive and nonspecific for MASLD [13,14,15]. Furthermore, as MASLD and metabolic syndrome share overlapping pathophysiological pathways, the observed association may be explained by central obesity and other metabolic components rather than vitamin D itself. Therefore, studies that have not included adequate adjustment for these confounders might fail to present an association independent of metabolic syndrome and its individual components. Moreover, most evidence to date comes from tertiary liver centers and advanced liver disease from other liver etiologies [16], leaving a gap in broader apparently heathy at-risk populations. Whether sufficient vitamin D levels mitigate the risk of steatosis in adults predisposed to MASLD therefore remains uncertain.

Importantly, beyond the presence or absence of vitamin D deficiency, the serum concentrations required for optimal hepatic health in MASLD have not been clearly defined. Recommended sufficiency threshold for serum vitamin D (>20 ng/mL) is primarily derived from skeletal outcomes and may not reflect the concentrations required for hepatic protection. MASLD develops in a pro-inflammatory and insulin-resistant milieu, conditions in which vitamin D metabolism, receptor signaling, and downstream effects may differ from those in the general population. As a result, individuals with vitamin D levels considered “sufficient” for bone health may still have concentrations that are suboptimal for preventing steatosis or fibrosis. Nevertheless, the potential existence of higher, MASLD-relevant vitamin D thresholds and dose–response relationships has not been systematically examined. We therefore hypothesized that higher serum vitamin D concentrations are associated with a lower prevalence of MASLD and fibrosis in a dose-dependent manner. Accordingly, we aimed to investigate the associations of serum vitamin D levels with MASLD in a cohort of adults at risk of MASLD, independent of metabolic syndrome and its individual components.

## 2. Materials and Methods

This cross-sectional study included adult participants attending the obesity and diabetes outpatient clinic of Istanbul University-Cerrahpaşa Hospital and an affiliated secondary-care internal medicine center, either for routine follow-up or initial evaluation of cardiometabolic conditions. Recruitment at both centers was conducted between February 2022 and December 2024. The study protocol was approved by the Istanbul University-Cerrahpaşa Clinical Research Ethics Committee (Ref. 2023/134) and the Istanbul Health Sciences University Clinical Research Ethics Committee (Ref. 2021/224). All study procedures were performed in accordance with the principles of the Declaration of Helsinki, and written informed consent was obtained from all participants prior to enrollment.

### 2.1. Study Population

Adults aged 18 to 80 years were eligible if they fulfilled at least one of the diagnostic criteria for MASLD at enrollment—namely central obesity, impaired glucose regulation, dyslipidemia, or elevated blood pressure. Additional requirements included the ability to give written informed consent and proficiency in speaking, reading, and writing Turkish. Individuals were excluded if they reported alcohol consumption exceeding 30 g/day for men or 20 g/day for women, had a history of hepatotoxic medication use, presented with alternative causes of chronic liver disease, had a prior diagnosis of hepatocellular carcinoma, or were pregnant or breastfeeding. The full set of exclusion criteria is available in Supplement S1.

### 2.2. Assessments

Following informed consent, each participant took part in a face-to-face study visit where anthropometric data were recorded, blood samples were obtained and MASLD evaluation was performed. Participants provided self-reported information about their education, income, smoking habits and lifestyle. Current medication and prior medical history were extracted from their medical records.

### 2.3. Clinical and Biochemical Data

Body weight was assessed to the nearest 0.05 kg using a calibrated bioelectrical impedance analyzer (Tanita, Tokyo, Japan) while participants were barefoot, lightly clothed, and in a fasting state. Standing height was measured to the closest 0.1 cm, and body mass index (BMI) was derived as weight in kilograms divided by height in meters squared (kg/m^2^). Waist circumference (WC) was obtained using a flexible tape, and central obesity was defined as WC > 80 cm in women and >94 cm in men [17]. Trained medical professionals gathered blood pressure readings from three measurements made with a standard mercury sphygmomanometer. Diabetes was defined according to the American Diabetes Association criteria and considered present if at least one of the following was observed: a history of diabetes, current use of oral hypoglycemic medication or insulin, or laboratory evidence of fasting plasma glucose ≥ 125 mg/dL or HbA1c ≥ 6.5% [18]. Metabolic syndrome was identified using the International Diabetes Federation definition [17].

After a 10 h overnight fast, venous blood samples were collected for biochemical analyses. Insulin resistance was estimated using the homeostasis model assessment (HOMA-IR). A HOMA-IR above 2.5 was considered as insulin resistance [19].

#### 2.3.1. Measurement of Serum Vitamin D Levels

Serum vitamin D was quantitatively measured by liquid chromatography tandem mass spectrometry [20]. Participants were categorized as having either vitamin D insufficiency (<20 ng/mL) or vitamin D sufficiency (≥20 ng/mL). Information on vitamin D supplementation was not consistently available across study sites and was therefore not included as a covariate in the analyses; serum vitamin D concentrations were used as an integrated measure of vitamin D status.

#### 2.3.2. MASLD and Fibrosis Assessment

MASLD and fibrosis were assessed using vibration-controlled transient elastography (VCTE) with controlled attenuation parameter (CAP) measurements, performed on a FibroScan device equipped with both M and XL probes. The M probe was applied by default, with automatic switching to the XL probe when prompted by the system. Valid examinations required a minimum three-hour fast, at least 10 reliable liver stiffness measurements (LSM), and an interquartile-to-median ratio below 30%. Steatosis was defined as CAP ≥ 248 dB/m, and significant fibrosis as LSM ≥ 8 kPa, in line with international clinical practice guidelines [21] and supported by previous evidence [22,23,24,25]. MASLD was diagnosed when liver steatosis, as determined by CAP, coexisted with at least one of five predefined cardiometabolic risk factors [21].

#### 2.3.3. Statistical Analysis

Continuous variables with normal distribution are expressed as mean ± SD and were assessed by unpaired Student’s *t*-test to report between-group differences. Categorical variables are expressed in absolute frequency and percentage.

##### Missing Data

Vitamin D levels were missing for 205 of the 1039 (20%) included participants. Differences between patients with and without Vitamin D measurements were assessed using unpaired Student’s t-test for continuous variables and Chi-square test for categorical variables. Factors associated with missing Vitamin D measurement were further examined using logistic regression. Missing vitamin D values were imputed using multiple imputation. The number of imputations was set to 40, approximately twice the percentage of missing data, in accordance with current guidelines for multiple imputation, which recommend that the number of imputations should be at least equal to the percentage of incomplete cases [26,27]. To strengthen the plausibility of the missing at random assumption, all factors associated with vitamin D missingness were included in the imputation model (for details, see Supplement S2).

##### Primary Analysis

The associations between Vitamin D status (examined as a dichotomous variable, by quartiles, and as a continuous measure) and MASLD and significant fibrosis were examined using logistic regression in both crude and multivariable models. The multivariable models were adjusted for site, season, age category, sex, income level, education level, Mediterranean diet adherence score, physical activity level score, alcohol, current smoking, obesity (defined by BMI), diabetes, hypertension, central obesity (defined by WC), dyslipidemia, insulin resistance and metabolic syndrome. Model assumptions were evaluated as follows: multicollinearity was assessed using variance inflation factors, with values > 10 indicating violation of this assumption; influential observations were defined as cases with Cook’s distance ≥ 1; and linearity of quantitative predictors was assessed by testing for significant quadratic terms. Sensitivity analyses were conducted using alternative adjustment strategies, including models excluding selected metabolic variables, to evaluate potential overadjustment. All statistical analyses were performed using SPSS 29.0.2.0 (IBM, New York, NY, USA) and *p* values < 0.05 were considered statistically significant.

## 3. Results

### 3.1. Baseline Characteristics

**Figure 1:**
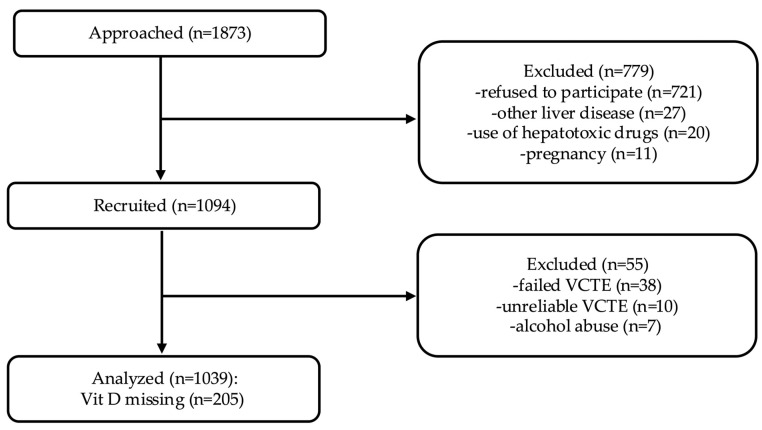
Study flowchart.

A total of 1,094 individuals were recruited for this study, of whom 834 had complete vitamin D levels (Figure 1). The mean age of the study population was 52.17 ± 12.99 years, and 51.6% were male. The mean BMI was 30.06 ± 5.77 kg/m^2^. MASLD and obesity were present in 54.7% (95% CI: 51.3, 58.0) and 45.1% (95% CI: 41.7, 48.5) of the subjects, respectively.

The mean serum vitamin D was 34.06 ± 17.28 ng/mL and the overall prevalence of vitamin D insufficiency was 18.8% (95% CI, 16.3, 21.6). Mean BMI, age, CAP, LSM, and serum TAG, FBG, HbA1c and HOMA-IR levels were significantly lower in subjects with sufficient serum vitamin D status. Prevalence of Vitamin D insufficiency was significantly higher among subjects with obesity than those without (76.3% vs. 23.7%, *p* = 0.001). Prevalence of vitamin D sufficiency did not show a statistically significant difference in subjects with and without diabetes, metabolic syndrome, hypertension and dyslipidemia. Table 1 shows the clinical and biochemical characteristics of the study population in relation to serum Vitamin D status.

### 3.2. Vitamin D and MASLD

**Figure 2:**
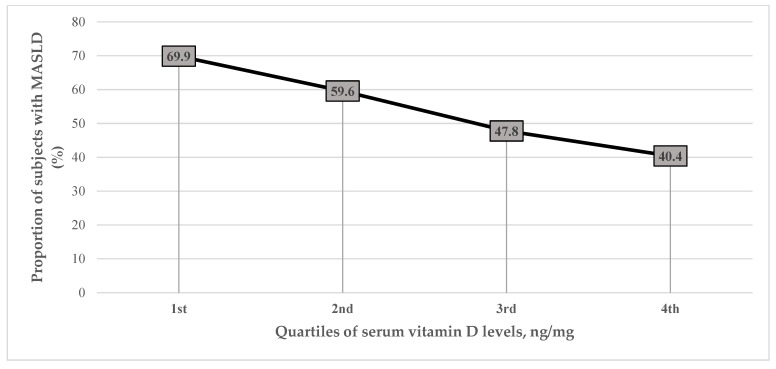
The proportion of subjects with MASLD according to quartiles of serum vitamin D levels: 1st: ≤22 ng/mL; 2nd: 23–32 ng/mL; 3rd: 33–43 ng/mL; 4th: ≥44 ng/mL.

Mean CAP (250.63 ± 52.40 vs. 276.73 ± 53.66, *p* < 0.001) and MASLD prevalence (51.4%, 95% CI: 47.6, 55.1 vs. 68.8%, 95% CI: 61.2, 75.5, *p* < 0.001) was significantly lower in patients with vitamin D sufficiency compared to subjects with insufficient vitamin D levels. Furthermore, the proportion of subjects with MASLD substantially decreased with increasing serum vitamin D levels from the lowest to highest quartiles (69.9, 59.6, 47.8 and 40.4%, *p* for linear trend = *p* < 0.001) (Figure 2). In line with this, vitamin D levels were significantly (*p* < 0.001) lower in patients with MASLD (30.16 ± 14.86 ng/mL) compared with those without (38.77 ± 18.78 ng/mL).

Sensitivity analyses excluding selected metabolic covariates yielded results consistent with the primary analyses (Supplement S5).

Table 2 shows the results of the logistic regression of Vitamin D and the odds of MASLD, based on multiple imputation of missing Vitamin D levels. When vitamin D was treated as a dichotomous variable (i.e., sufficient vs. insufficient), sufficient serum vitamin D status was associated with 53% lower odds of MASLD (OR = 0.47, 95% CI: 0.33–0.67, *p* < 0.001) in the crude model. After adjusting for potential confounders, this association attenuated to a 40% reduction in odds (OR = 0.60, 95% CI: 0.36–1.03), and the *p*-value increased to 0.063, indicating that the adjusted association was no longer statistically significant, although it remained clinically relevant. When serum vitamin D levels were categorized into quartiles (i.e., ≤22 ng/mL, 23–32 ng/mL, 33–43 ng/mL, and ≥44 ng/mL), participants in the highest quartile (i.e., ≥44 ng/mL) had a 72% lower odds of MASLD in the crude model (OR = 0.28, 95% CI: 0.19–0.41, *p* < 0.001), and a 61% lower odds in the adjusted model (OR = 0.39, 95% CI: 0.21–0.71, *p* = 0.002), compared with those in the lowest quartile (i.e., ≤22 ng/mL). When serum vitamin D was treated as a continuous variable, each 1 ng/mL increase was associated with a 3% lower odds of MASLD (OR = 0.97, 95% CI: 0.96–0.98, *p* < 0.001) in the crude model. After adjusting for potential confounders, the association remained statistically significant, with a slightly smaller effect of 2% lower odds per 1 ng/mL increase (OR = 0.98, 95% CI: 0.96, 0.99, *p* < 0.001). Complete-case analyses were also performed, yielding similar effect estimates (Supplement S3 and S4). Assessment of model assumptions revealed no violations. Sensitivity analyses excluding selected metabolic covariates yielded results consistent with the primary analyses (Supplement S5).

Table 3 shows the results of logistic regression of Vitamin D status and the odds of MASLD in different sub-groups. Vitamin D sufficiency was associated with a clinically relevant reduction in the odds of MASLD across all subgroups, with the magnitude of effect varying: 70% lower odds in participants with metabolic syndrome (adjusted OR = 0.30, 95% CI 0.12–0.73), 58% lower in those with hypertension (adjusted OR = 0.42, 95% CI 0.20–0.92), 57% lower in those with obesity (adjusted OR = 0.43, 95% CI 0.18–1.02), 56% lower in those with central obesity (adjusted OR = 0.44, 95% CI 0.23–0.82), 49% lower in those with diabetes (adjusted OR = 0.51, 95% CI 0.20–1.30), 37% lower in those with dyslipidemia (adjusted OR = 0.63, 95% CI 0.35–1.14), and 35% lower in those with insulin resistance (adjusted OR = 0.65, 95% CI 0.31–1.34). Across all subgroups, the effect consistently suggested lower odds of MASLD with Vitamin D sufficiency. The highest vitamin D quartile (≥44 ng/mL) was associated with a clinically relevant reduction in the odds of MASLD in all subgroups. Sensitivity analyses excluding influential outliers in the metabolic syndrome and diabetes subgroups did not materially alter these findings. Results of the complete case analysis are provided in Supplementary S6.

In age-stratified analyses, vitamin D sufficiency was not independently associated with MASLD after multivariable adjustment. In contrast, higher vitamin D concentrations were associated with a more pronounced reduction in the odds of MASLD among older adults compared with younger participants (Supplement S7).

### 3.3. Vitamin D and Fibrosis

Mean LSM was significantly lower in patients with sufficient vitamin D levels compared to those with insufficient levels (5.34 ± 2.14 vs. 5.94 ± 2.60, *p* = 0.008). The crude model showed that sufficient serum vitamin D status was associated with 54% lower odds of significant fibrosis (adjusted OR = 0.46, 95% CI: 0.28–0.76, *p* = 0.002). This association remained significant and of similar magnitude after adjustment for potential confounders (adjusted OR = 0.48, 95% CI: 0.25–0.92, *p* = 0.026). When vitamin D was categorized into quartiles, a significant association was observed only for the highest vitamin D quartile compared with the lowest quartile (crude OR = 0.43, 95% CI: 0.21–0.87, *p* = 0.018); however, this association was substantially attenuated after adjustment for potential confounders (adjusted OR = 0.81, 95% CI 0.34–1.94, *p* = 0.64). Similarly, the inverse association between serum vitamin D levels (as ng/mL) and the odds of fibrosis observed in the crude model was not maintained in the multivariable model (Supplement S8 and S9).

## 4. Discussion

In this cross-sectional study of adults at risk of MASLD, vitamin D sufficiency, defined by serum vitamin D > 20 ng/mL, was associated with a 40% reduction in the adjusted odds of hepatic steatosis, although this association did not reach conventional statistical significance (*p* = 0.063). In addition, a clear and significant dose–response relationship was observed, with participants in the highest vitamin D quartile (i.e., ≥44 ng/mL) showing the lowest odds of MASLD compared with those in the lowest quartile (i.e., ≤22 ng/mL). Vitamin D sufficiency was also associated with lower odds of significant fibrosis, but this protective effect was not consistently observed when Vitamin D was categorized into quartiles after adjustment for confounders.

The role of vitamin D in liver-related conditions has attracted considerable interest in recent years, with particular focus on MASLD. An inverse association between the circulating levels of Vitamin D and presence of MASLD has been pointed out by population-level studies [7,14,28,29,30,31,32,33,34] as well as meta-analyses [4,5,35]. However, despite relatively large sample sizes, many of these investigations relied on serum biomarkers [14,28,33] and ultrasonography [29,30,31,32], both of which limit generalizability. VCTE, by contrast, offers greater sensitivity in detecting low-grade steatosis. Consistent with our results, a previous VCTE-based study reported lower odds of MASLD for higher vitamin D levels than for the lowest quartile of vitamin D in a dose-dependent manner [6], while another reported reduced odds of VCTE-defined MASLD among participants with sufficient vitamin D levels [7]. In line with these findings, our results indicate a dose-dependent effect, with progressively higher vitamin D levels linked to lower MASLD odds instead of a threshold effect, where sufficient vitamin D status confers protection. Notably, only a minority of the prior studies adequately adjusted for metabolic syndrome, despite its strong overlap with both MASLD and vitamin D status, conditions that are increasingly prevalent and often coexist in clinical practice. Our findings indicate that vitamin D may influence MASLD independently of metabolic syndrome, supporting some earlier reports [11,13,31,36]. Comparable associations have also been observed in case–control studies [13,37,38], although other studies found no significant relationship [8,12,39]. Studies that failed to demonstrate an association may reflect methodological constraints such as small sample sizes, use of serum transaminases as surrogates for MASLD, or limited control for confounders. The present study adds clarity by addressing these limitations through its relatively large sample size, multicenter recruitment, use of VCTE rather than biochemical markers, and adjustment for metabolic syndrome. Together, these methodological strengths enhance the reliability of our findings and support the role of vitamin D as an independent correlate of MASLD.

The optimal serum vitamin D level for metabolic and hepatic health remains to be established. The currently accepted cutoff for sufficiency (≥20 ng/mL) is primarily based on skeletal outcomes and may not accurately reflect the threshold relevant for extra-skeletal effects, including hepatic fat accumulation and inflammation [40]. Earlier recommendations proposing a higher cutoff of 30 ng/mL to achieve broader health benefits were later retracted, as they were not supported by sufficient clinical outcome data [41]. Nevertheless, several population-based studies have suggested that metabolic benefits may continue to grow up to 30–40 ng/mL, whereas higher levels may not provide additional protection and could even exhibit a plateau effect [4,7,35]. Similarly, a recent meta-analysis summarizing dose–response curves across multiple health outcomes identified the lowest risk for metabolic diseases at serum vitamin D levels of approximately 40 ng/mL [42]. In our study, serum vitamin D levels above 33 ng/mL were broadly associated with additional protection against MASLD, suggesting that the conventional 20 ng/mL threshold may underestimate the optimal range for hepatic outcomes. Furthermore, concentrations exceeding 44 ng/mL conferred further protection in all considered sub-groups. From a clinical perspective, while serum vitamin D cannot be considered a diagnostic marker for MASLD, these findings suggest that vitamin D status may be relevant for hepatic risk stratification in adults with cardiometabolic risk factors, particularly when interpreted alongside established metabolic and imaging-based assessments. The overall effect of serum vitamin D in the whole study population (adjusted OR = 0.98, *p* < 0.001) suggests that the protective effect of higher serum vitamin D concentrations may be broadly universal. Furthermore, the observed interaction by age suggests that the association between vitamin D status and MASLD might be modified by life stage. While higher vitamin D concentrations were associated with lower MASLD prevalence across all age groups, the association was strongest and most consistent in participants older than 60. This pattern may partly be explained by longer exposure to metabolic risk factors and increased susceptibility to hepatic fat accumulation with advancing age. Notably, across all age groups, vitamin D levels exceeding conventional sufficiency thresholds were required to observe independent protective associations, underscoring the limitations of binary deficiency definitions in MASLD research and highlighting the potential clinical relevance of vitamin D assessment in metabolically at-risk populations. However, given the cross-sectional nature of this study, the results should not be interpreted as evidence to support vitamin D supplementation for MASLD prevention or treatment. Rather, they highlight the need for prospective and interventional studies to determine whether improving vitamin D status can favorably influence MASLD development or progression. Our findings, together with earlier observations, support the notion that maintaining serum vitamin D above the deficiency range may be important for hepatic health, yet the precise concentration that maximizes metabolic and hepatoprotective effects is still uncertain. Future studies should aim to identify biological thresholds specific to hepatic endpoints and determine whether vitamin D optimization beyond general sufficiency confers incremental benefits in MASLD prevention and management.

Our study showed a significant but not a dose-dependent association between vitamin D levels and significant fibrosis. The relationship between liver fibrosis and vitamin D is a relatively less studied topic, and the results obtained from previous evidence are therefore conflicting. In line with our findings, a recent analysis of NHANES 2017–2018 data reported that subjects with significant fibrosis had lower serum vitamin D levels and that subjects with sufficient vitamin D levels had a reduced risk of liver fibrosis [43]. Other authors that analyzed the same dataset similarly reported lower odds of significant liver fibrosis for participants with sufficient vitamin D levels [7]. Wan et al., on the other hand, reported no association between vitamin D levels and liver fibrosis even though vitamin D insufficiency was linked to higher odds of MASLD after adjustment for other confounders [32]. Another VCTE study with a sample of apparently healthy subjects (n = 1202) reported no significant difference in LSM between vitamin D-deficient and -sufficient groups [6]. However, the authors did not report any further analysis of the association between vitamin D levels and liver fibrosis. Our study showed that vitamin D sufficiency is associated with lower odds of significant fibrosis, but this effect did not extend across quartiles of vitamin D distribution, supporting the presence of a threshold effect rather than a dose–response relationship. Similarly, the crude inverse association between serum vitamin D levels and the odds of fibrosis dissipated after adjustment for metabolic syndrome and its components, indicating that the relationship is at least partly explained by metabolic confounding. Taken together, our results support the notion that correcting deficiency may be important for liver health, but additional increases above sufficiency do not appear to confer further protection against fibrosis. Because the prevalence of significant fibrosis in our study population was relatively low, these results should be interpreted as exploratory, and further research in populations with more advanced liver disease is warranted to better define the optimal vitamin D status in the context of fibrosis.

Multiple mechanisms should be considered to interpret our findings. Vitamin D influences adipose tissue biology by modulating adipogenesis and promoting a more favorable adipokine profile, thereby improving systemic insulin sensitivity [44]. It also attenuates chronic low-grade inflammation through suppression of pro-inflammatory cytokines and regulation of innate immune responses, processes central to MASLD pathogenesis [45]. Moreover, vitamin D signaling has been implicated in hepatic stellate cell activation and fibrogenesis, suggesting a potential role in fibrosis development [16]. In our study, the association between vitamin D and hepatic steatosis was more pronounced than that with fibrosis. This discrepancy may reflect that steatosis is an earlier and more reversible manifestation of MASLD, whereas fibrosis develops gradually and is influenced by a more complex interplay of genetic, metabolic, and environmental factors. Consequently, vitamin D deficiency may act as an initiating factor in lipid accumulation and inflammation, but its direct contribution to fibrotic remodeling may be less apparent or masked by stronger determinants such as long-standing metabolic stress or genetic susceptibility. Furthermore, MASLD is characterized by a long, often asymptomatic natural history, with hepatic steatosis typically developing years before progression to fibrosis. Longitudinal studies have shown that disease progression is heterogeneous and influenced by cumulative metabolic burden, aging, and inflammatory exposure, rather than by a clearly defined disease onset [46]. As a result, disease duration is difficult to ascertain in population-based or metabolically at-risk cohorts, particularly in the absence of prior imaging or histological data. In this context, age may serve as a pragmatic proxy for cumulative exposure to metabolic risk factors and subclinical liver injury. This may partly explain the stronger and more consistent associations between higher vitamin D concentrations and MASLD observed in middle-aged and older participants in the present study. Another explanation is the relatively low number of participants with significant fibrosis in our cohort, which may have limited statistical power to detect an association. In advanced liver disease, impaired hepatic hydroxylation capacity can lead to markedly reduced circulating vitamin D levels, particularly in late stages of cirrhosis. Indeed, studies that defined MASLD by biopsy and included participants with more advanced disease stages reported stronger associations between vitamin D insufficiency and fibrosis [10,36,47]. Taken together, these observations suggest that although vitamin D possesses well-described anti-fibrogenic properties, its role in fibrogenesis in MASLD populations remains incompletely understood.

The most important strength of this study is the use of the established definition of MASLD, i.e., the co-existence of hepatic steatosis on imaging together with cardiometabolic disturbances. Another strength is the use of sensitive and accurate modalities to diagnose both MASLD and vitamin D insufficiency. Nevertheless, several limitations should be acknowledged. First, the cross-sectional design precludes assessment of chronological patterns, making it impossible to determine whether low vitamin D levels preceded MASLD onset. Thus, while causality cannot be inferred, the results highlight a strong and independent association between lower serum vitamin D levels and the presence of MASLD after adjustment for multiple confounders. Second, although VCTE is extensively validated for quantifying liver steatosis and fibrosis, liver biopsy remains the gold standard. However, its invasive nature, susceptibility to sampling error, and risk of complications limit its feasibility in large population-based studies. Third, the results of the primary analyses using multiple imputation are valid under the (untestable) missing at random assumption. To enhance the plausibility of this assumption, variables associated with missing Vitamin D levels were included in the imputation model. Finally, the lack of detailed information on vitamin D supplementation, including dose and duration, represents a limitation, although serum vitamin D levels reflect the net effect of supplementation, diet, and endogenous synthesis.

In conclusion, this study demonstrated that vitamin D insufficiency is associated with higher odds of MASLD in a dose-dependent manner, independent of metabolic syndrome and its components. Its impact on fibrosis, on the other hand, is yet to be fully elucidated. In addition to traditional case–control and cross-sectional studies, bidirectional Mendelian randomization studies and well-designed interventional clinical trials are warranted to understand the potential casual role of Vitamin D in the pathogenesis of MASLD.

## Figures and Tables

**Table 1:** Clinical characteristics of the study population according to serum Vitamin D status, Vitamin D sufficient: >20 mg/dL.

	All (n = 834)	Vitamin D Sufficient (n = 677)	Vitamin D Insufficient (n = 157)	*p* Value
Demographics				
Age	52.17 ± 12.99	53.41 ± 12.68	46.80 ± 13.05	<0.001
Sex (%Male)	51.6%	51.8%	50.3%	NS
BMI (kg/m^2^)	30.06 ± 5.77	29.39 ± 5.14	32.95 ± 7.28	<0.001
Lifestyle				
Alcohol (%)	43.5%	44.5%	39.5%	NS
Smoker (%)	30.1%	27.5%	41.4%	0.001
MEDAS	6.39 ± 2.11	6.56 ± 2.11	5.66 ± 1.97	<0.001
PAL	992.06 ± 993.04	1066.78 ± 1009	669.85 ± 849.43	<0.001
Biochemical measurements				
Total cholesterol (mg/dL)	197.09 ± 49.24	197.46 ± 50.70	195.47 ± 42.48	NS
HDL-cholesterol (mg/dL)	50.17 ± 14.35	51.04 ± 14.59	46.45 ± 12.66	<0.001
LDL-cholesterol (mg/dL)	119.92 ± 42.52	119.39 ± 43.99	122.21 ± 35.48	NS
TAG (mg/dL)	152.86 ± 102.01	146.73 ± 92.55	179.29 ± 132.60	0.004
FBS (mg/dL)	101.44 ± 38.68	98.77 ± 30.78	112.97 ± 60.97	<0.001
HbA1c (%) (n = 815)	5.90 ± 1.15	5.823 ± 1.04	6.23 ± 1.51	0.002
HOMA-IR	3.81 ± 4.22	3.61 ± 3.85	4.66 ± 5.47	0.024
25(OH)D (ng/mL)	34.06 ± 17.28	38.71 ± 15.79	14.02 ± 3.97	<0.001
ALT (UI/l)	26.21 ± 17.42	26.11 ± 17.24	26.64 ± 18.26	NS
AST (UI/l)	22.0 ± 12.26	22.25 ± 12.47	21.37 ± 11.29	NS
GGT (UI/l)	28.80 ± 30.79	28.58 ± 32.21	29.79 ± 23.79	NS
Liver measurements				
CAP (dB/m)	255.54 ± 53.59	250.63 ± 52.40	276.73 ± 53.66	<0.001
LSM (kPa)	5.46 ± 2.24	5.34 ± 2.14	5.94 ± 2.60	0.008
FIB-4	0.98 ± 0.62	1.03 ± 0.65	0.77 ± 0.42	<0.001
FAST	0.11 ± 0.14	0.10 ± 0.12	0.12 ± 0.15	NS
Medical history				
MASLD (%)	54.7 (51.3, 58.0)	51.4 (47.6, 55.1)	68.8 (61.2, 75.5)	<0.001
Obesity (%)	45.1 (41.7, 48.5)	42.4 (38.7, 46.1)	56.7 (48.9, 64.2)	0.001
Diabetes (%)	40.3 (37.0, 43.7)	38.8 (35.2, 42.6)	46.5 (38.9, 54.3)	NS
Hypertension (%)	61.9 (58.5, 65.1)	63.4 (59.7, 66.9)	55.4 (47.6, 63.0)	NS
Dyslipidemia (%)	80.3 (77.5, 82.9)	81.2 (78.1, 84.0)	76.4 (69.2, 82.4)	NS
Metabolic syndrome (%)	53.7 (50.3, 57.1)	52.1 (48.4, 55.9)	60.5 (52.7, 67.8)	NS

Data are presented as mean ± standard deviation and frequencies (95 CI%); *p* < 0.05 considered statistically significant. Abbreviations: ALT, alanine transaminase; AST, aspartate transaminase; BMI, body mass index; CAP, controlled attenuation parameter; FAST, FibroScan-AST score; FBG, fasting blood glucose; FIB-4, fibrosis-4 index; FI, fibrosis index; GGT; Gamma-glutamyl transferase, HDL, high-density lipoprotein; HOMA-IR, homeostatic model assessment of insulin resistance; LDL, low-density lipoprotein; LSM, liver stiffness measurement; TAG, triglycerides; TC, total cholesterol.

**Table 2:** Univariable and multivariable logistic regression models showing the effect of Vitamin D on the odds of MASLD defined by CAP *≥* 248 (based on multiple imputation, n = 1039).

	Crude Model		Adjusted Model ^a^	
	OR (95% CI)	*p*	OR (95% CI)	*p*
Vitamin D sufficiency (≥20 ng/mL)	0.47 (0.33, 0.67)	<0.001	0.60 (0.36, 1.03)	0.063
Quartiles of serum Vitamin D				
1st (≤22 ng/mL)	1 (reference)		1 (reference)	
2nd (23–32 ng/mL)	0.61 (0.42, 0.89)	0.011	0.65 (0.39, 1.09)	0.102
3rd (33–43 ng/mL)	0.42 (0.28, 0.62)	<0.001	0.43 (0.25, 0.75)	0.003
4th (≥44 ng/mL)	0.28 (0.19, 0.41)	<0.001	0.39 (0.21, 0.71)	0.002
Serum vitamin D (ng/mL)	0.97 (0.96, 0.98)	<0.001	0.98 (0.96, 0.99)	<0.001

^a^ Adjusted for: site, vit D season, age category, education level, income level, alcohol (y/n), gender, smoker (y/n), Mediterranean diet adherence score, physical activity level score, obesity (y/n), diabetes (y/n), insulin resistance (y/n), hypertension (y/n), dyslipidemia (y/n), metabolic syndrome (y/n), central obesity (y/n).

**Table 3:** Univariable and multivariable logistic regression models showing the effect of Vitamin D on the odds of MASLD defined by CAP *≥* 248. Results based on multiple imputation.

	Crude Model		Adjusted Model	
	OR (95% CI)	*p*	OR (95% CI)	*p*
**Obesity (n = 504)**				
Vitamin D sufficiency (≥20 ng/mL)	0.57 (0.30, 1.08)	0.085	0.43 (0.18, 1.02)	0.057
Quartiles of serum Vitamin D				
1st (≤22 ng/mL)	1 (reference)		1 (reference)	
2nd (23–32 ng/mL)	0.48 (0.25, 0.92)	0.026	0.44 (0.20, 0.98)	0.045
3rd (33–43 ng/mL)	0.40 (0.20, 0.79)	0.009	0.31 (0.12, 0.76)	0.010
4th (≥44 ng/mL)	0.74 (0.31, 1.74)	0.484	0.46 (0.15, 1.38)	0.165
Serum vitamin D (ng/mL)	0.99 (0.97, 1.00)	0.130	0.98 (0.96, 0.99)	0.035
Adjusted for: site, age, sex, education, income, alcohol, smoker, VitD season, DM, IR, HT, Dyslipidemia, MetS, highWC, MEDAS, PAL				
**Diabetes (n = 411)**				
Vitamin D sufficiency (≥20 ng/mL)	0.42 (0.22, 0.81)	0.009	0.51 (0.20, 1.30)	0.157
Quartiles of serum Vitamin D				
1st (≤22 ng/mL)	1 (reference)		1 (reference)	
2nd (23–32 ng/mL)	0.59 (0.29, 1.16)	0.127	0.49 (0.20, 1.24)	0.131
3rd (33–43 ng/mL)	0.50 (0.26, 0.98)	0.042	0.49 (0.19, 1.28)	0.147
4th (≥44 ng/mL)	0.20 (0.10, 0.40)	<0.001	0.25 (0.09, 0.70)	0.009
Serum vitamin D (ng/mL)	0.96 (0.95, 0.98)	<0.001	0.96 (0.94, 0.99)	0.002
Adjusted for: site, age, sex, education, income, alcohol, smoker, VitD season, obesity, IR, HT, Dyslipidemia, MetS, highWC, MEDAS, PAL				
**Metabolic Syndrome (n = 562)**				
Vitamin D sufficiency (≥20 ng/mL)	0.25 (0.11, 0.53)	<0.001	0.30 (0.12, 0.73)	0.008
Quartiles of serum Vitamin D				
1st (≤22 ng/mL)	1 (reference)		1 (reference)	
2nd (23–32 ng/mL)	0.29 (0.14, 0.62)	0.001	0.31 (0.14, 0.72)	0.006
3rd (33–43 ng/mL)	0.17 (0.08, 0.36)	<0.001	0.19 (0.08, 0.45)	<0.001
4th (≥44 ng/mL)	0.16 (0.08, 0.37)	<0.001	0.19 (0.08, 0.51)	<0.001
Serum vitamin D (ng/mL)	0.97 (0.95, 0.98)	<0.001	0.97 (0.95, 0.98)	<0.001
Adjusted for: site, VitD season, age, sex, education, income, alcohol, smoker, obesity, DM, IR, HT, Dyslipidemia, MEDAS, PAL				
**Hypertension (n = 599)**				
Vitamin D sufficiency (≥20 ng/mL)	0.32 (0.18, 0.56)	<0.001	0.42 (0.20, 0.92)	0.029
Quartiles of serum Vitamin D				
1st (≤22 ng/mL)	1 (reference)		1 (reference)	
2nd (23–32 ng/mL)	0.46 (0.25, 0.82)	0.008	0.43 (0.19, 0.93)	0.032
3rd (33–43 ng/mL)	0.29 (0.17, 0.51)	<0.001	0.28 (0.13, 0.60)	<0.001
4th (≥44 ng/mL)	0.18 (0.10, 0.30)	<0.001	0.29 (0.13, 0.65)	0.003
Serum vitamin D (ng/mL)	0.96 (0.95, 0.97)	<0.001	0.97 (0.96, 0.99)	0.002
Adjusted for: site, VitD season, age, sex, education, income, alcohol, smoker, obesity, DM, IR, Dyslipidemia, MetS, highWC, MEDAS, PAL				
**Dyslipidemia (n = 837)**				
Vitamin D sufficiency (≥20 ng/mL)	0.48 (0.32, 0.72)	<0.001	0.63 (0.35, 1.14)	0.123
Quartiles of serum Vitamin D				
1st (≤22 ng/mL)	1 (reference)		1 (reference)	
2nd (23–32 ng/mL)	0.63 (0.40, 0.98)	0.040	0.68 (0.39,1.22)	0.202
3rd (33–43 ng/mL)	0.41 (0.27, 0.63)	<0.001	0.43 (0.23, 0.79)	0.007
4th (≥44 ng/mL)	0.28 (0.18, 0.43)	<0.001	0.43 (0.22, 0.84)	0.013
Serum vitamin D (ng/mL)	0.97 (0.96, 0.98)	<0.001	0.98 (0.96, 0.99)	0.001
Adjusted for: site, VitD season, age, sex, education, income, alcohol, smoker, obesity, DM, IR, MetS, highWC, MEDAS, PAL				
**Central Obesity (n = 780)**				
Vitamin D sufficiency (≥20 ng/mL)	0.44 (0.27, 0.71)	<0.001	0.44 (0.23, 0.82)	0.010
Quartiles of serum Vitamin D				
1st (≤22 ng/mL)	1 (reference)		1 (reference)	
2nd (23–32 ng/mL)	0.52 (0.32, 0.84)	0.008	0.53 (0.29, 0.95)	0.034
3rd (33–43 ng/mL)	0.40 (0.24, 0.64)	<0.001	0.33 (0.17, 0.63)	<0.001
4th (≥44 ng/mL)	0.41 (0.24, 0.69)	<0.001	0.39 (0.19, 0.81)	0.012
Serum vitamin D (ng/mL)	0.98 (0.97, 0.99)	<0.001	0.98 (0.96, 0.99)	0.003
Adjusted for: site, VitD season, age, sex, education, income, alcohol, smoker, obesity, DM, IR, Dyslipidemia, MetS, MEDAS, PAL.				
**Insulin Resistance (n = 591)**				
Vitamin D sufficiency (≥20 ng/mL)	0.54 (0.32, 0.92)	0.024	0.65 (0.31, 1.34)	0.240
Quartiles of serum Vitamin D				
1st (≤22 ng/mL)	1 (reference)		1 (reference)	
2nd (23–32 ng/mL)	0.54 (0.30, 0.96)	0.034	0.52 (0.26, 1.05)	0.070
3rd (33–43 ng/mL)	0.58 (0.32, 1.03)	0.064	0.70 (0.32, 1.51)	0.359
4th (≥44 ng/mL)	0.31 (0.17, 0.56)	<0.001	0.43 (0.19, 0.98)	0.045
Serum vitamin D (ng/mL)	0.97 (0.96, 0.99)	<0.001	0.98 (0.96, 0.99)	0.030
Adjusted for: site, VitD season, age, sex, education, income, alcohol, smoker, obesity, DM, Dyslipidemia, MetS, high WC, MEDAS, PAL.				

## Data Availability

The original contributions presented in this study are included in the article/Supplementary Material. Further inquiries can be directed to the corresponding author.

## Supplementary Materials

The following supporting information can be downloaded at: https://www.mdpi.com/article/10.3390/nu18040599/s1. S1: Inclusion and exclusion criteria; S2: Multiple imputation supplement; S3: Univariable logistic regression models showing the effect of Vitamin D on the odds of MASLD; S4: Multivariable logistic regression models showing the effect of Vitamin D on the odds of MASLD; S5: Univariable and multivariable logistic regression models showing the effect of Vitamin D on the odds of MASLD in subgroups; S6: Univariable logistic regression models showing the effect of Vitamin D on the odds of significant fibrosis defined by LSM ≥ 8 kPa; S7: Multivariable logistic regression models showing the effect of Vitamin D on the odds of significant fibrosis defined by LSM ≥ 8 kPa.
