# Supplementary material for: Vitamin D Sufficiency Revisited: Evidence of a Dose–Response Effect for MASLD in Adults at Risk
Source: Nutrients. 2026 Feb 11;18(4):599. doi: 10.3390/nu18040599 (PMC12943758; doi:10.3390/nu18040599)
Supplement: Supplementary file 1 [file nutrients-18-00599-s001.zip › nutrients-4068929-supplementary.pdf]

SUPPLEMENTARY INFORMATION

Supplement S1. Inclusion and exclusion criteria

Inclusion Criteria:

- Able to understand and sign the informed consent
- Able to speak Turkish
- Between 18-80 years
- Having one of the following conditions:
- BMI ≥25 kg/m² or waist circumference ≥94 cm in men, ≥80 cm in women
- Impaired glucose tolerence: HbA1c 5.7-6.4% or fasting plasma glucose 100-125 mg/dl
- Type 2 diabetes mellitus: HbA1c ≥6.5% or fasting plasma glucose ≥126 mg/dl
- Treatment for type 2 diabetes
- ≥130/85 mmHg or treatment for hypertension
- *Triglycerides* ≥150 mg/dl or HDL ≤39 mg/dl in men, ≤50 mg/dl in women or lipid-lowering treatment

Exclusion Criteria:

- Excessive alcohol use (more than 20 g/day for women and 30g/day for men= >2 glasses alcohol/day for women and >3 glasses for men)
- Other liver diseases: Hepatitis B virus, Hepatitis C virus, autoimmune hepatitis, primary biliary cirrhosis, hemochromatosis, Wilson's disease, Alpha 1 antitrypsin deficiency
- Secondary causes for steatosis: disorders of lipid metabolism, HCV Genotype 3, total parental nutrition, severe surgical weight loss, medications (amiodarone, tamoxifen, methotrexate, corticosteroids and HAART), lean steatosis, Celiac disease, environmental toxicity
- Pregnancy and breastfeeding.
- Diagnosis of liver cirrhosis and/or hepatocellular carcinoma.
- Current diagnosis of extrahepatic malignancy(s) or prior diagnosis within last 5 years.

Supplement S2. Multiple Imputation Supplement

Following the reporting guidelines of Sterne et al. (2009) about missing data, we show here some important details about the missing data and the method to handle them:

1. Number of missing values for each variable of interest:

	Vitamin D	Vitamin D Season	HbA1c
Number of complete cases	834	834	898
Number missing values	205	205	141

2. Number of cases with complete data: 815/1039
3. Differences between complete and incomplete data:

Characteristic	Number of complete cases	Number of missing values	Vitamin D missing (n=205)	Vitamin D available (n=834)	p
Age (years)	1039	0	49.97 (12.75)	52.17 (13.00)	0.029
BMI (kg/m²)	1039	0	32.49 (5.81)	30.06 (5.77)	<0.001
MEDAS score	1039	0	5.97 (1.91)	6.39 (2.11)	0.006
PAL:<br>inactive<br>active<br>highly active	1039	0	 143 (28%)<br>58 (12.7%)<br>4 (5.6%)	 368 (72%)<br>398 (87.3%)<br>68 (94.4%)	<0.001
Total cholesterol	1039	0	192.25 (40.03)	197.09 (49.24)	0.140
HDL (mg/dL)	1039	0	41.25 (13.15)	50.17 (14.35)	<0.001
LDL (mg/dL)	1039	0	102.80 (34.19)	119.92 (42.52)	<0.001
TAG (mg/dl)	1039	0	156.83 (101.16)	152.86 (102.01)	0.617
FBS (mg/dl)	1039	0	100.69 (38.75)	101.44 (38.67)	0.804
Hba1c (%)	898	141	6.04 (1.43)	5.90 (1.15)	0.296
HOMA-IR	1039	0	4.22 (3.19)	3.81 (4.22)	0.191
ALT (UI/l)	1039	0	16.20 (14.90)	26.21 (17.42)	<0.001
AST (UI/l)	1039	0	16.51 (9.91)	22.09 (12.26)	<0.001
GGT (UI/l)	1039	0	26.49 (32.93)	28.80 (30.79)	0.341
CAP (dB/m)	1039	0	278.40 (53.77)	255.54 (53.58)	<0.001
LSM (kPa)	1039	0	5.52 (1.64)	5.45 (2.24)	0.687
FIB-4	1039	0	.80 (.43)	.98 (.62)	<0.001
FAST	1039	0	.07 (.12)	.11 (.13)	<0.001
Education (years)	1039	0	10.96 (4.8)	12.41 (4.67)	<0.001
Site:<br>Pax clinic<br>Ctf	1039<br>580<br>459	0	 9 (1.6%)<br>196 (42.7%)	 571 (98.4%)<br>263 (57.3%)	<0.001
Sex (male)	1039	0	83 (16.2%)	430 (83.8%)	0.005
Alcohol	1039	0	47 (11.5%)	363 (88.5%)	<0.001
Smoker:<br>Never<br>Ex-smoker<br>Current	1039<br>691<br>44<br>304	0	 151 (21.9%)<br>1 (2.3%)<br>53 (17.4%)	 540 (78.1%)<br>43 (97.7%)<br>251 (82.6%)	0.003
MASLD	1039	0	141 (23.6%)	456 (76.4%)	<0.001

Obesity	1039	0	128 (25.4%)	376 (74.6%)	<0.001
Diabetes	1039	0	75 (18.2%)	336 (81.8%)	0.331
Hypertension	1039	0	83 (13.9%)	516 (86.1%)	<0.001
Dyslipidaemia	1039	0	167 (20%)	670 (80%)	0.715
Metabolic syndrome	1039	0	114 (20.3%)	448 (79.7%)	0.626
Income	1039	0			0.01
Very low	21		2 (9.5%)	19 (90.5%)	
Low	126		20 (15.9%)	106 (84.1%)	
Middle	323		68 (21.1%)	255 (78.9%)	
Middle high	242		64 (26.4%)	178 (73.6%)	
High	327		51 (15.6%)	276 (84.4%)	
Insulin resistance	1039	0	145 (24.5%)	446 (75.5%)	<0.001
Central obesity	1039	0	175 (22.4%)	605 (77.6%)	<0.001
Significant fibrosis	1039	0	28 (26.2%)	79 (73.8%)	0.077

Data are presented as mean (standard deviation), or count (%)

4. Logistic regression with missing vitamin D level (yes/no) as outcome and all the variables in the table above as independent variables (with the exception of vitamin D itself). From the starting model, predictors were removed with backward elimination based on the likelihood ratio test, with significance level 0.20.

Starting model

Variable	Wald P-value	Exp(B)	95% CI LB	95% CI UB
Site=CTF	<.001	52.176	18.625	146.167
Age	.299	1.017	.985	1.050
Sex (Male)	.725	.880	.432	1.792
BMI	.566	.976	.900	1.059
Alcohol	.946	1.028	.461	2.293
Smoking	0.596			
Ex-smoker		.727	1.491	.158
Smoker		.346	.702	.336
MEDAS score	.174	.900	.773	1.048
PAL score	.534			
		.894	.957	.500
		.262	.290	.033
TC	<.001	1.034	1.019	1.050
HDL	.006	.961	.934	.988
LDL	<.001	.960	.944	.975
TAG	.005	.995	.992	.999
FBS	.789	.998	.987	1.010
HbA1c	.276	1.253	.835	1.882
HOMA	.817	1.010	.931	1.094
GGT	.046	.965	.931	.999
CAP	.090	1.046	.993	1.101
LSM	.637	.997	.983	1.010
FIB4	.534	.997	.986	1.007
FAST	.113	.831	.661	1.045
MASLD	.559	.787	.353	1.757
obesity	.260	21.983	.102	4738.880

Diabetes	.765	1.176	.406	3.405
Hypertension	.293	.594	.225	1.567
Dyslipidemia	.018	.391	.179	.852
ALT	.202	.633	.313	1.279
AST	.336	.667	.293	1.520
Metabolic syndrome	.870	.929	.385	2.243
Education	.020	1.091	1.014	1.174
Income	.366			
Low		2.441	.231	25.844
Middle		2.215	.228	21.481
Middle High		1.577	.155	16.021
High		.983	.092	10.526
Insulin resistance	<.001	4.099	1.794	9.368
Central obesity	.103	2.537	.830	7.759
Significant Fibrosis	.706	.781	.217	2.812
Constant	.070	.010		

Final model

Variable	Wald P-value	Exp(B)	95% CI LB	95% CI UB
Site=CTF	<.001	45.779	18.922	110.752
MEDAS score	.193	.910	.791	1.049
TC	<.001	1.032	1.018	1.046
HDL	.002	.963	.940	.987
LDL	<.001	.961	.946	.975
TAG	.001	.995	.992	.998
HbA1c	.177	1.183	.927	1.510
ALT	.048	.972	.945	1.000
AST	.012	1.047	1.010	1.085
LSM	.076	.865	.737	1.015
Obesity	.061	.486	.228	1.033
Diabetes	.006	.374	.184	.758
Education	.059	1.061	.998	1.128
Insulin resistance	<.001	3.763	1.772	7.993
Central obesity	.080	2.312	.905	5.907
constant	<.001	.007		

5. Missing data were handled with multiple imputation
    a. Software and key setting: SPSS version 28, using the fully conditional specification approach. Predictive-mean matching was used for continuous variables (i.e., vitamin D, HbA1c) and logistic regression for binary variables (i.e., vitamin D season).
    b. Imputation model: the variables included in the imputation model can be classified in the following groups: (i) the outcomes (i.e., MASLD, significant fibrosis) and their continuous version (i.e., CAP and LSM), (ii) all the variables in the logistic regression models: vitamin D (continuous), site, vitamin D season, age, education, income, alcohol use, sex, smoking, Mediterranean diet adherence (MEDAS) score, categories of physical activity level (PAL),

obesity, diabetes, insulin resistance, hypertension, dyslipidaemia, metabolic syndrome, central obesity, and (iii) variables, not part of groups (i)-(ii), that were shown, at significance level 0.20, to be associated with missing vitamin D either based on the final logistic regression in 4. or the univariable tests in 3.: TC, HDL, LDL, TAG, HbA1c, ALT, AST, HOMA-IR, FIB4, FAST, BMI. All variables were treated as predictors only, with the exception of vitamin D, vitamin D season, and HbA1c that were both outcome and predictor in the imputation model. No interactions were included in the imputation model.

c. Number of imputed datasets: 40.

Supplement S3. Univariable logistic regression models showing the effect of Vitamin D on the odds of MASLD

	**P-value**	**OR**	**95% CI LB**	**95% CI UB**
Complete Case Analyses (N=834)				
Vitamin D sufficiency	<.001	.480	.332	.695
Multiple Imputation (N=1039)				
Vitamin D sufficiency	<.001	.470	.327	.673

	**P-value**	**OR**	**95% CI LB**	**95% CI UB**
Complete Case Analyses (N=834)				
Vitamin D quartiles	<.001			
1st (≤ 22 ng/ml)	reference			
2nd (23-32 ng/ml)	.010	.598	.403	.886
3rd (33- 43 ng/ml)	<.001	.410	.276	.609
4th (≥ 44 ng/ml)	<.001	.279	.187	.417
Multiple Imputation (N=1039)				
Vitamin D quartiles	<.001 (median p-value)			
1st (≤ 22 ng/ml)	reference			
2nd (23-32 ng/ml)	.011	.610	.416	.894
3rd (33- 43 ng/ml)	<.001	.419	.284	.618
4th (≥ 44 ng/ml)	<.001	.275	.186	.407

	**P-value**	**OR**	**95% CI LB**	**95% CI UB**
Complete Case Analyses (N=834)				
Vitamin D	<.001	.969	.960	.978
Multiple Imputation (N=1039)				
Vitamin D	<.001	.969	.960	.977

NB: SPSS does not provide for the pooled analysis after MI the overall p-value, so the one provided under MI is a median of all 40 p-values.

Supplement S4. Multivariable logistic regression models showing the effect of Vitamin D on the odds of MASLD

	**P-value**	**OR**	**95% CI LB**	**95% CI UB**
Complete Case Analyses (N=834)				
Vitamin D sufficiency	.054	.600	.357	1.009
Multiple Imputation (N=1039)				
Vitamin D sufficiency	.063	.604	.355	1.027

	**P-value**	**OR**	**95% CI LB**	**95% CI UB**
Complete Case Analyses (N=834)				
Vitamin D quartiles	0.002			
1st (≤ 22 ng/ml)	reference			
2nd (23-32 ng/ml)	.041	.573	.336	.979
3rd (33- 43 ng/ml)	<.001	.383	.218	.673
4th (≥ 44 ng/ml)	<.001	.350	.192	.638
Multiple Imputation (N=1039)				
Vitamin D quartiles	<0.01 (median p-value)			
1st (≤ 22 ng/ml)	reference			
2nd (23-32 ng/ml)	.102	.653	.392	1.089
3rd (33- 43 ng/ml)	.003	.430	.246	.750
4th (≥ 44 ng/ml)	.002	.388	.213	.706

	**P-value**	**OR**	**95% CI LB**	**95% CI UB**
Complete Case Analyses (N=834)				
Vitamin D	<.001	.974	.962	.987
Multiple Imputation (N=1039)				
Vitamin D	<.001	.976	.963	.989

Adjusted for: site, season, age (categorical), education (categorical), income (categories), alcohol, sex, current smoker (binary), MEDAS score, PAL score/1000 (NOTE: this rescales the OR of PAL, but does not affect the other ORs), obesity, diabetes, insulin resistance, hypertension, dyslipidemia, metabolic syndrome, central obesity

For the three models above the following assumptions are met:

- Linearity: for all categorical predictors and for MEDAS (score), PAL score/1000 (continuous), vitamin D (continuous)
- No multicollinearity: all VIFs <10
- No influential outliers: all Cook's distances<1

Supplement S5. Sensitivity analyses of showing the effect of Vitamin D sufficiency (> 20 ng/mL) on the odds of MASLD, complete case analysis

Initial Full Model

	**P-value**	**OR**	**95% CI LB**	**95% CI UB**
Complete Case Analyses (N=834)				
Vitamin D sufficiency	.054	.600	.357	1.009

Adjusted for: site, season, age (categorical), education (categorical), income (categories), alcohol, sex, current smoker (binary), MEDAS score, PAL score/1000 (NOTE: this rescales the OR of PAL, but does not affect the other ORs), obesity, diabetes, insulin resistance, hypertension, dyslipidemia, metabolic syndrome, central obesity

Sensitivity Analysis Model 1 (excluding insulin resistance, metabolic syndrome, central obesity)

	**P-value**	**OR**	**95% CI LB**	**95% CI UB**
Complete Case Analyses (N=834)				
Vitamin D sufficiency	.056	.623	.383	1.011

Adjusted for: site, season, age (categorical), education (categorical), income (categories), alcohol, sex, current smoker (binary), MEDAS score, PAL score/1000 (NOTE: this rescales the OR of PAL, but does not affect the other ORs), obesity, diabetes, hypertension, dyslipidemia

Sensitivity Analysis Model 2 (excluding obesity, insulin resistance, metabolic syndrome)

	**P-value**	**OR**	**95% CI LB**	**95% CI UB**
Complete Case Analyses (N=834)				
Vitamin D sufficiency	.059	.616	.373	1.019

Adjusted for: site, season, age (categorical), education (categorical), income (categories), alcohol, sex, current smoker (binary), MEDAS score, PAL score/1000 (NOTE: this rescales the OR of PAL, but does not affect the other ORs), central obesity, diabetes, hypertension, dyslipidemia

Sensitivity Analysis Model 3 (excluding diabetes, central obesity, hypertension, dyslipidemia)

	**P-value**	**OR**	**95% CI LB**	**95% CI UB**
Complete Case Analyses (N=834)				
Vitamin D sufficiency	.103	.679	.411	1.121

Adjusted for: site, season, age (categorical), education (categorical), income (categories), alcohol, sex, current smoker (binary), MEDAS score, PAL score/1000 (NOTE: this rescales the OR of PAL, but does not affect the other ORs), obesity, metabolic syndrome, insulin resistance

Supplement S6. Univariable and multivariable logistic regression models showing the effect of Vitamin D on the odds of MASLD in subgroups

Obesity - Crude models

	**P-value**	**OR**	**95% CI LB**	**95% CI UB**
Complete Case Analyses (N=376)				
Vitamin D sufficiency	.064	.532	.273	1.038
Multiple Imputation (N=504)				
Vitamin D sufficiency	.085	.567	.297	1.081

	**P-value**	**OR**	**95% CI LB**	**95% CI UB**
Complete Case Analyses (N=376)				
Vitamin D quartiles	.007			
1st (≤ 22 ng/ml)	reference			
2nd (23-32 ng/ml)	.007	.382	.191	.765
3rd (33- 43 ng/ml)	.002	.325	.160	.658
4th (≥ 44 ng/ml)	.433	.699	.286	1.710
Multiple Imputation (N=504)				
Vitamin D quartiles	0.018 (median p-value)			
1st (≤ 22 ng/ml)	reference			
2nd (23-32 ng/ml)	.026	.476	.248	.915
3rd (33- 43 ng/ml)	.009	.401	.203	.792
4th (≥ 44 ng/ml)	.484	.735	.310	1.742

	**P-value**	**OR**	**95% CI LB**	**95% CI UB**
Complete Case Analyses (N=376)				
Vitamin D	.170	.988	.972	1.005
Multiple Imputation (N=504)				
Vitamin D	.130	.987	.971	1.004

Obesity - adjusted models

	**P-value**	**OR**	**95% CI LB**	**95% CI UB**
Complete Case Analyses (N=376)				
Vitamin D sufficiency	.045	.406	.169	.978
Multiple Imputation (N=504)				
Vitamin D sufficiency	.057	.434	.184	1.024

	**P-value**	**OR**	**95% CI LB**	**95% CI UB**
Complete Case Analyses (N=376)				
Vitamin D quartiles	.033			
1st (≤ 22 ng/ml)	reference			

2nd (23-32 ng/ml)	.033	.390	.164	.928
3rd (33- 43 ng/ml)	.004	.250	.098	.637
4th (≥ 44 ng/ml)	.135	.427	.140	1.304
Multiple Imputation (N=504)				
Vitamin D quartiles	0.008 (median p-value)			
1st (≤ 22 ng/ml)	reference			
2nd (23-32 ng/ml)	.045	.444	.201	.981
3rd (33- 43 ng/ml)	.010	.305	.124	.755
4th (≥ 44 ng/ml)	.165	.460	.154	1.376

	**P-value**	**OR**	**95% CI LB**	**95% CI UB**
Complete Case Analyses (N=376)				
Vitamin D	.047	.978	.957	1.000
Multiple Imputation (N=504)				
Vitamin D	.035	.977	.956	.998

Adjusted for: site, season, age (categorical), education (categorical), income (categories), alcohol, sex, current smoker (binary), MEDAS score, PAL score/1000 (NOTE: this rescales the OR of PAL, but does not affect the other ORs), obesity, diabetes, insulin resistance, hypertension, dyslipidemia, metabolic syndrome, central obesity

Diabetes - crude models

	**P-value**	**OR**	**95% CI LB**	**95% CI UB**
Complete Case Analyses (N=336)				
Vitamin D sufficiency	.011	.419	.214	.820
Multiple Imputation (N=411)				
Vitamin D sufficiency	.009	.417	.216	.805

	**P-value**	**OR**	**95% CI LB**	**95% CI UB**
Complete Case Analyses (N=336)				
Vitamin D quartiles	<.001			
1st (≤ 22 ng/ml)	reference			
2nd (23-32 ng/ml)	.139	.585	.287	1.190
3rd (33- 43 ng/ml)	.063	.522	.263	1.035
4th (≥ 44 ng/ml)	<.001	.209	.105	.417
Multiple Imputation (N=411)				
Vitamin D quartiles	XXX (median p-value)			
1st (≤ 22 ng/ml)	reference			
2nd (23-32 ng/ml)	.127	.585	.294	1.164
3rd (33- 43 ng/ml)	.042	.501	.257	.975
4th (≥ 44 ng/ml)	<.001	.202	.103	.399

	**P-value**	**OR**	**95% CI LB**	**95% CI UB**
Complete Case Analyses (N=336)				

Vitamin D	<.001	.962	.948	.978
Multiple Imputation (N=411)				
Vitamin D	<.001	.962	.947	.978

Diabetes - adjusted models

	P-value	OR	95% CI LB	95% CI UB
Complete Case Analyses (N=336)				
Vitamin D sufficiency	.107	.458	.177	1.184
Multiple Imputation (N=411)				
Vitamin D sufficiency	.157	.512	.202	1.295

	P-value	OR	95% CI LB	95% CI UB
Complete Case Analyses (N=336)				
Vitamin D quartiles	.054			
1st (≤ 22 ng/ml)	reference			
2nd (23-32 ng/ml)	.108	.453	.172	1.189
3rd (33- 43 ng/ml)	.193	.516	.190	1.398
4th (≥ 44 ng/ml)	.007	.219	.073	.658
Multiple Imputation (N=411)				
Vitamin D quartiles	0.044 (median p-value)			
1st (≤ 22 ng/ml)	reference			
2nd (23-32 ng/ml)	.131	.494	.197	1.236
3rd (33- 43 ng/ml)	.147	.493	.189	1.284
4th (≥ 44 ng/ml)	.009	.245	.085	.704

	P-value	OR	95% CI LB	95% CI UB
Complete Case Analyses (N=336)				
Vitamin D	.002	.963	.940	.986
Multiple Imputation (N=411)				
Vitamin D	.002	.964	.942	.987

Adjusted for: site, season, age (categorical), education (categorical), income (categories), alcohol, sex, current smoker (binary), MEDAS score, PAL score/1000 (NOTE: this rescales the OR of PAL, but does not affect the other ORs), obesity, diabetes, insulin resistance, hypertension, dyslipidemia, metabolic syndrome, central obesity

Metabolic syndrome - crude models

	P-value	OR	95% CI LB	95% CI UB
Complete Case Analyses (N=448)				
Vitamin D sufficiency	<.001	.186	.079	.439
Multiple Imputation (N=562)				
Vitamin D sufficiency	<.001	.246	.114	.533

	P-value	OR	95% CI LB	95% CI UB
Complete Case Analyses (N=448)				
Vitamin D quartiles	<.001			
1st (≤ 22 ng/ml)	reference			
2nd (23-32 ng/ml)	<.001	.198	.086	.457
3rd (33- 43 ng/ml)	<.001	.120	.053	.270
4th (≥ 44 ng/ml)	<.001	.130	.056	.303
Multiple Imputation (N=562)				
Vitamin D quartiles	0.044 (median p-value)			
1st (≤ 22 ng/ml)	reference			
2nd (23-32 ng/ml)	.001	.290	.135	.621
3rd (33- 43 ng/ml)	<.001	.174	.084	.360
4th (≥ 44 ng/ml)	<.001	.175	.082	.374

	P-value	OR	95% CI LB	95% CI UB
Complete Case Analyses (N=448)				
Vitamin D (ng/mL)	<.001	.965	.951	.979
Multiple Imputation (N=562)				
Vitamin D (ng/mL)	<.001	.965	.952	.978

Metabolic syndrome - adjusted models

	P-value	OR	95% CI LB	95% CI UB
Complete Case Analyses (N=448)				
Vitamin D sufficiency	.002	.202	.074	.547
Multiple Imputation (N=562)				
Vitamin D sufficiency	.008	.300	.123	.734

	P-value	OR	95% CI LB	95% CI UB
Complete Case Analyses (N=448)				
Vitamin D quartiles	<.001			
1st (≤ 22 ng/ml)	reference			
2nd (23-32 ng/ml)	<.001	.191	.075	.490
3rd (33- 43 ng/ml)	<.001	.117	.045	.303
4th (≥ 44 ng/ml)	<.001	.132	.048	.368
Multiple Imputation (N=562)				

Vitamin D quartiles	0.001 (median p-value)			
1st (≤ 22 ng/ml)	reference			
2nd (23-32 ng/ml)	.006	.312	.135	.719
3rd (33- 43 ng/ml)	<.001	.192	.082	.453
4th (≥ 44 ng/ml)	<.001	.199	.078	.507

	**P-value**	**OR**	**95% CI LB**	**95% CI UB**
Complete Case Analyses (N=448)				
Vitamin D	<.001	.966	.949	.983
Multiple Imputation (N=562)				
Vitamin D	<.001	.968	.951	.985

Adjusted for: site, season, age (categorical), education (categorical), income (categories), alcohol, sex, current smoker (binary), MEDAS score, PAL score/1000 (NOTE: this rescales the OR of PAL, but does not affect the other ORs), obesity, diabetes, insulin resistance, hypertension, dyslipidemia, metabolic syndrome, central obesity

Hypertension - crude models

	**P-value**	**OR**	**95% CI LB**	**95% CI UB**
Complete Case Analyses (N=516)				
Vitamin D sufficiency	<.001	.292	.160	.534
Multiple Imputation (N=599)				
Vitamin D sufficiency	<.001	.315	.179	.556

	**P-value**	**OR**	**95% CI LB**	**95% CI UB**
Complete Case Analyses (N=516)				
Vitamin D quartiles	<.001			
1st (≤ 22 ng/ml)	reference			
2nd (23-32 ng/ml)	.007	.431	.234	.794
3rd (33- 43 ng/ml)	<.001	.275	.155	.488
4th (≥ 44 ng/ml)	<.001	.170	.096	.298
Multiple Imputation (N=599)				
Vitamin D quartiles	XXXX (median p-value)			
1st (≤ 22 ng/ml)	reference			
2nd (23-32 ng/ml)	.008	.455	.254	.815
3rd (33- 43 ng/ml)	<.001	.294	.170	.510
4th (≥ 44 ng/ml)	<.001	.177	.103	.303

	**P-value**	**OR**	**95% CI LB**	**95% CI UB**
Complete Case Analyses (N=516)				
Vitamin D	<.001	.962	.951	.974
Multiple Imputation (N=599)				

Vitamin D	<.001	.962	.951	.973

Hypertension - adjusted models

	**P-value**	**OR**	**95% CI LB**	**95% CI UB**
Complete Case Analyses (N=516)				
Vitamin D sufficiency	.018	.376	.167	.846
Multiple Imputation (N=599)				
Vitamin D sufficiency	.029	.423	.196	.915

	**P-value**	**OR**	**95% CI LB**	**95% CI UB**
Complete Case Analyses (N=516)				
Vitamin D quartiles	.002			
1st (≤ 22 ng/ml)	reference			
2nd (23-32 ng/ml)	.013	.352	.155	.802
3rd (33- 43 ng/ml)	<.001	.230	.104	.508
4th (≥ 44 ng/ml)	.001	.255	.111	.589
Multiple Imputation (N=599)				
Vitamin D quartiles	0.002 (median p-value)			
1st (≤ 22 ng/ml)	reference			
2nd (23-32 ng/ml)	.032	.425	.194	.930
3rd (33- 43 ng/ml)	<.001	.276	.130	.589
4th (≥ 44 ng/ml)	.003	.286	.126	.649

	**P-value**	**OR**	**95% CI LB**	**95% CI UB**
Complete Case Analyses (N=516)				
Vitamin D	.002	.974	.958	.990
Multiple Imputation (N=599)				
Vitamin D	.002	.974	.958	.990

Adjusted for: site, season, age (categorical), education (categorical), income (categories), alcohol, sex, current smoker (binary), MEDAS score, PAL score/1000 (NOTE: this rescales the OR of PAL, but does not affect the other ORs), obesity, diabetes, insulin resistance, hypertension, dyslipidemia, metabolic syndrome, central obesity

Dyslipidemia - crude models

	P-value	OR	95% CI LB	95% CI UB
Complete Case Analyses (N=670)				
Vitamin D sufficiency	<.001	.470	.303	.728
Multiple Imputation (N=837)				
Vitamin D sufficiency	<.001	.477	.317	.720

	P-value	OR	95% CI LB	95% CI UB
Complete Case Analyses (N=670)				
Vitamin D quartiles	<.001			
1st (≤ 22 ng/ml)	reference			
2nd (23-32 ng/ml)	.026	.590	.371	.938
3rd (33- 43 ng/ml)	<.001	.377	.238	.598
4th (≥ 44 ng/ml)	<.001	.264	.166	.418
Multiple Imputation (N=837)				
Vitamin D quartiles	XXX (median p-value)			
1st (≤ 22 ng/ml)	reference			
2nd (23-32 ng/ml)	.040	.628	.403	.979
3rd (33- 43 ng/ml)	<.001	.409	.266	.627
4th (≥ 44 ng/ml)	<.001	.277	.179	.430

	P-value	OR	95% CI LB	95% CI UB
Complete Case Analyses (N=670)				
Vitamin D	<.001	.968	.959	.978
Multiple Imputation (N=837)				
Vitamin D	<.001	.969	.959	.978

Dyslipidemia - adjusted models

	P-value	OR	95% CI LB	95% CI UB
Complete Case Analyses (N=670)				
Vitamin D sufficiency	.083	.584	.318	1.072
Multiple Imputation (N=837)				
Vitamin D sufficiency	.123	.626	.345	1.136

	P-value	OR	95% CI LB	95% CI UB
Complete Case Analyses (N=670)				
Vitamin D quartiles	.008			
1st (≤ 22 ng/ml)	reference			
2nd (23-32 ng/ml)	.062	.556	.301	1.029
3rd (33- 43 ng/ml)	.001	.353	.186	.670
4th (≥ 44 ng/ml)	.004	.371	.189	.729
Multiple Imputation (N=837)				

Vitamin D quartiles	0.004 (median p-value)			
1st (≤ 22 ng/ml)	reference			
2nd (23-32 ng/ml)	.202	.686	.385	1.224
3rd (33- 43 ng/ml)	.007	.428	.232	.792
4th (≥ 44 ng/ml)	.013	.429	.220	.835

	**P-value**	**OR**	**95% CI LB**	**95% CI UB**
Complete Case Analyses (N=670)				
Vitamin D	<.001	.975	.962	.989
Multiple Imputation (N=837)				
Vitamin D	.001	.977	.963	.991

Adjusted for: site, season, age (categorical), education (categorical), income (categories), alcohol, sex, current smoker (binary), MEDAS score, PAL score/1000 (NOTE: this rescales the OR of PAL, but does not affect the other ORs), obesity, diabetes, insulin resistance, hypertension, dyslipidemia, metabolic syndrome, central obesity

Central obesity - crude models

	**P-value**	**OR**	**95% CI LB**	**95% CI UB**
Complete Case Analyses (N=605)				
Vitamin D sufficiency	<.001	.385	.233	.639
Multiple Imputation (N=780)				
Vitamin D sufficiency	<.001	.440	.273	.710

	**P-value**	**OR**	**95% CI LB**	**95% CI UB**
Complete Case Analyses (N=605)				
Vitamin D quartiles	<.001			
1st (≤ 22 ng/ml)	reference			
2nd (23-32 ng/ml)	.002	.448	.273	.736
3rd (33- 43 ng/ml)	<.001	.340	.205	.562
4th (≥ 44 ng/ml)	<.001	.370	.216	.635
Multiple Imputation (N=780)				
Vitamin D quartiles	XXX (median p-value)			
1st (≤ 22 ng/ml)	reference			
2nd (23-32 ng/ml)	.008	.519	.321	.839
3rd (33- 43 ng/ml)	<.001	.394	.243	.638
4th (≥ 44 ng/ml)	<.001	.408	.242	.688

	**P-value**	**OR**	**95% CI LB**	**95% CI UB**
Complete Case Analyses (N=605)				
Vitamin D	<.001	.978	.967	.989
Multiple Imputation (N=780)				
Vitamin D	<.001	.978	.967	.989

Central obesity - adjusted models

	P-value	OR	95% CI LB	95% CI UB
Complete Case Analyses (N=605)				
Vitamin D sufficiency	.003	.383	.202	.728
Multiple Imputation (N=780)				
Vitamin D sufficiency	.010	.438	.234	.819

	**P-value**	**OR**	**95% CI LB**	**95% CI UB**
Complete Case Analyses (N=605)				
Vitamin D quartiles	.002			
1st (≤ 22 ng/ml)	reference			
2nd (23-32 ng/ml)	.008	.425	.227	.796
3rd (33- 43 ng/ml)	<.001	.272	.140	.530
4th (≥ 44 ng/ml)	.004	.347	.167	.720
Multiple Imputation (N=780)				
Vitamin D quartiles	0.001 (median p-value)			
1st (≤ 22 ng/ml)	reference			
2nd (23-32 ng/ml)	.034	.527	.291	.954
3rd (33- 43 ng/ml)	<.001	.330	.173	.630
4th (≥ 44 ng/ml)	.012	.396	.193	.813

	**P-value**	**OR**	**95% CI LB**	**95% CI UB**
Complete Case Analyses (N=605)				
Vitamin D	.002	.977	.963	.992
Multiple Imputation (N=780)				
Vitamin D	.003	.977	.963	.992

Adjusted for: site, season, age (categorical), education (categorical), income (categories), alcohol, sex, current smoker (binary), MEDAS score, PAL score/1000 (NOTE: this rescales the OR of PAL, but does not affect the other ORs), obesity, diabetes, insulin resistance, hypertension, dyslipidemia, metabolic syndrome, central obesity

Insulin resistance - crude models

	**P-value**	**OR**	**95% CI LB**	**95% CI UB**
Complete Case Analyses (N=446)				
Vitamin D sufficiency	.038	.557	.321	.967
Multiple Imputation (N=591)				
Vitamin D sufficiency	.024	.541	.318	.921

	**P-value**	**OR**	**95% CI LB**	**95% CI UB**
Complete Case Analyses (N=446)				
Vitamin D quartiles	.002			
1st (≤ 22 ng/ml)	reference			

2nd (23-32 ng/ml)	.018	.493	.275	.885
3rd (33- 43 ng/ml)	.085	.591	.324	1.076
4th (≥ 44 ng/ml)	<.001	.315	.173	.572
Multiple Imputation (N=591)				
Vitamin D quartiles	XXX (median p-value)			
1st (≤ 22 ng/ml)	reference			
2nd (23-32 ng/ml)	.034	.539	.304	.955
3rd (33- 43 ng/ml)	.064	.578	.324	1.031
4th (≥ 44 ng/ml)	<.001	.311	.173	.559

	**P-value**	**OR**	**95% CI LB**	**95% CI UB**
Complete Case Analyses (N=446)				
Vitamin D	<.001	.974	.963	.986
Multiple Imputation (N=591)				
Vitamin D	<.001	.974	.962	.985

Insulin resistance - adjusted models

	**P-value**	**OR**	**95% CI LB**	**95% CI UB**
Complete Case Analyses (N=446)				
Vitamin D sufficiency	.248	.640	.300	1.365
Multiple Imputation (N=591)				
Vitamin D sufficiency	.240	.647	.313	1.339

	**P-value**	**OR**	**95% CI LB**	**95% CI UB**
Complete Case Analyses (N=446)				
Vitamin D quartiles	.049			
1st (≤ 22 ng/ml)	reference			
2nd (23-32 ng/ml)	.019	.407	.192	.865
3rd (33- 43 ng/ml)	.363	.688	.308	1.539
4th (≥ 44 ng/ml)	.020	.372	.161	.856
Multiple Imputation (N=591)				
Vitamin D quartiles	0.038 (median p-value)			
1st (≤ 22 ng/ml)	reference			
2nd (23-32 ng/ml)	.070	.522	.258	1.054
3rd (33- 43 ng/ml)	.359	.696	.320	1.513
4th (≥ 44 ng/ml)	.045	.434	.192	.980

	**P-value**	**OR**	**95% CI LB**	**95% CI UB**
Complete Case Analyses (N=446)				
Vitamin D	.024	.981	.964	.997
Multiple Imputation (N=591)				

Vitamin D	.030	.982	.965	.998

Adjusted for: site, season, age (categorical), education (categorical), income (categories), alcohol, sex, current smoker (binary), MEDAS score, PAL score/1000 (NOTE: this rescales the OR of PAL, but does not affect the other ORs), obesity, diabetes, insulin resistance, hypertension, dyslipidemia, metabolic syndrome, central obesity

Supplement S7. Univariable and multivariable logistic regression models showing the effect of Vitamin D on the odds of MASLD, stratified by age group

		**Crude Model**		**Adjusted Model[a]**	
		**OR (95% CI)**	**p**	**OR (95% CI)**	**p**
**Age: 18-47**	**Vitamin D sufficiency (≥20 ng/ml)**	0.490 (0.289,0.830)	0.008	0.631 (0.282,1.411)	0.262
	**Quartiles of serum Vitamin D**				
	**1st (≤ 22 ng/ml)**	1 (reference)		1 (reference)	
	**2nd (23-32 ng/ml)**	0.761 (0.409,1.418)	0.390	0.749 (0.289, 1.946)	0.553
	**3rd (33- 43 ng/ml)**	0.323 (0.163, 0.637)	0.001	0.259 (0.092, 0.727)	0.010
	**4th (≥ 44 ng/ml)**	0.435 (0.212, 0.893)	0.023	0.988 (0.314, 3.109)	0.983
	**Serum vitamin D (ng/mL)**	0.969 (0.953, 0.985)	<0.001	0.984 (0.960, 1.008)	0.190
**48-59**	**Vitamin D sufficiency (≥20 ng/ml)**	0.314 (0.145, 0.681)	0.003	0.448 (0.155,1.300)	0.140
	**Quartiles of serum Vitamin D**				
	**1st (≤ 22 ng/ml)**	1 (reference)		1 (reference)	
	**2nd (23-32 ng/ml)**	0.448 (0.214, 0.941)	0.034	0.324 (0.104, 1.010)	0.052
	**3rd (33- 43 ng/ml)**	0.331 (0.161, 0.681)	0.003	0.421 (0.146, 1.216)	0.110
	**4th (≥ 44 ng/ml)**	0.152 (0.072, 0.321)	<0.001	0.208 (0.065, 0.667)	0.008
	**Serum vitamin D (ng/mL)**	0.962 (0.946, 0.977)	<0.001	0.967 (0.944, 0.991)	0.007
**60-80**	**Vitamin D sufficiency (≥20 ng/ml)**	0.358 (0.148, 0.863)	0.004	0.421 (0.126, 1.407)	0.160
	**Quartiles of serum Vitamin D**				
	**1st (≤ 22 ng/ml)**	1 (reference)		1 (reference)	
	**2nd (23-32 ng/ml)**	0.482 (0.199, 1.168)	0.106	0.402 (0.129, 1.250)	0.115
	**3rd (33- 43 ng/ml)**	0.286 (0.127, 0.641)	0.002	0.216 (0.073, 0.640)	0.006
	**4th (≥ 44 ng/ml)**	0.206 (0.094, 0.448)	<0.001	0.247 (0.085, 0.7189	0.010
	**Serum vitamin D (ng/mL)**	0.963 (0.947, 0.979)	<0.001	0.968 (0.946, 0.992)	0.008

Supplement S8. Univariable logistic regression models showing the effect of Vitamin D on the odds of significant fibrosis defined by LSM≥8 kPa

	P-value	OR	95% CI LB	95% CI UB
Complete Case Analyses (N=834)				
Vitamin D sufficiency	.001	.428	.258	.710
Multiple Imputation (N=1039)				
Vitamin D sufficiency	.002	.456	.275	.757

	P-value	OR	95% CI LB	95% CI UB
Complete Case Analyses (N=834)				
Vitamin D quartiles	.119			
1st (≤ 22 ng/ml)	reference			
2nd (23-32 ng/ml)	.121	.607	.323	1.141
3rd (33- 43 ng/ml)	.498	.814	.448	1.478
4th (≥ 44 ng/ml)	.026	.450	.223	.907
Multiple Imputation (N=1039)				
Vitamin D quartiles	0.038 (median p-value)			
1st (≤ 22 ng/ml)	reference			
2nd (23-32 ng/ml)	.118	.616	.336	1.132
3rd (33- 43 ng/ml)	.309	.735	.405	1.332
4th (≥ 44 ng/ml)	.018	.426	.210	.866

	P-value	OR	95% CI LB	95% CI UB
Complete Case Analyses (N=834)				
Vitamin D	.007	.978	.963	.994
Multiple Imputation (N=1039)				
Vitamin D	.005	.977	.961	.993

Supplement S9. Multivariable logistic regression models showing the effect of Vitamin D on the odds of significant fibrosis defined by LSM≥8 kPa

	**P-value**	**OR**	**95% CI LB**	**95% CI UB**
Complete Case Analyses (N=834)				
Vitamin D sufficiency	.004	.379	.194	.738
Multiple Imputation (N=1039)				
Vitamin D sufficiency	.026	.476	.247	.916

	**P-value**	**OR**	**95% CI LB**	**95% CI UB**
Complete Case Analyses (N=834)				
Vitamin D quartiles	.702			
1st (≤ 22 ng/ml)	reference			
2nd (23-32 ng/ml)	.359	.710	.342	1.475
3rd (33- 43 ng/ml)	.814	1.091	.526	2.263
4th (≥ 44 ng/ml)	.693	.838	.347	2.020
Multiple Imputation (N=1039)				
Vitamin D quartiles	0.511 (median p-value)			
1st (≤ 22 ng/ml)	reference			
2nd (23-32 ng/ml)	.250	.664	.330	1.335
3rd (33- 43 ng/ml)	.933	.969	.464	2.023
4th (≥ 44 ng/ml)	.639	.812	.339	1.943

	**P-value**	**OR**	**95% CI LB**	**95% CI UB**
Complete Case Analyses (N=834)				
Vitamin D	.281	.989	.969	1.009
Multiple Imputation (N=1039)				
Vitamin D	.292	.989	.969	1.010

Adjusted for: site, season, age (categorical), education (categorical), income (categories), alcohol, sex, current smoker (binary), MEDAS score, PAL categories, obesity, diabetes, insulin resistance, hypertension, dyslipidemia, metabolic syndrome, central obesity

For the models above the following assumptions are met:

- Linearity: for all categorical predictors (including PAL categories) and for MEDAS (score), vitamin D (continuous)
- No multicollinearity: all VIFs <10
- No influential outliers: all Cook's distances<1, except for the complete case analysis where one influential outlier was found.
